# The impact of diaper design on mitigating known causes of diaper dermatitis

**DOI:** 10.1111/pde.13680

**Published:** 2018-08-30

**Authors:** Jennifer Gustin, Roger Gibb, David Maltbie, Donald Roe, Susana Waimin Siu

**Affiliations:** ^1^ Procter & Gamble Cincinnati Ohio

**Keywords:** breastfed stool, diaper dermatitis, neonatal, quality of life, skin barrier, stool management

## Abstract

**Background:**

Diapers play a critical role in infant health. In addition to providing sanitary methods of disposing of urine and feces, they can also directly impact skin health. Prolonged exposure to wetness and fecal matter has been shown to be a key driver of diaper dermatitis. This study sought to evaluate how diaper construction can affect absorption of stool.

**Methods:**

Methods included laboratory testing of stool absorbency as well as an at‐home diaper evaluation study, which examined a diaper's ability to keep fecal matter from the skin. Breastfed infant stool was given special consideration, as its properties make it difficult to contain.

**Results:**

Laboratory results demonstrated that a meshlike aperture diaper was better able to absorb fecal matter. The at‐home diaper evaluation study confirmed that a meshlike aperture diaper design resulted in fewer instances of stool being present on skin during diaper changes.

**Conclusion:**

Diapers with a meshlike aperture topsheet may represent a better way to mitigate known causes of diaper dermatitis through their superior ability to absorb fecal matter.

## 1 INTRODUCTION

Disposable diapers represent an essential consumer product in modern life. Diaper technology has advanced considerably and can contribute to improved quality of life by imparting dryness, hygiene, leakage control, and comfort.^1^ Diapers are worn in close contact with the skin and, as such, can play a key role in managing skin health. Modern‐day disposable diapers are constructed with absorbency features that lock moisture away from the skin.^2^

The absorption and sequestering of fecal matter away from the skin are essential to protecting infant skin health. When in contact with skin, bile salts, fecal enzymes, and other irritants found in stool can begin to break down the protective top layers of skin, which can induce diaper dermatitis. Prolonged stool contact with an infant's skin may also further exacerbate irritation, especially when the outer layers of skin are already compromised.^3^

Managing this microenvironment within the diaper by preventing exposure to elements that cause skin breakdowns is essential to reducing the risk of rash and irritation.^4^ One of the most important features of a diaper is the topsheet, the part of the diaper in immediate contact with an infant. The topsheet plays a key role in mitigating stool contact with the skin and can play a key role in skin‐related conditions, like diaper dermatitis. Previous research has demonstrated the role that diaper materials have in reducing the incidence of diaper dermatitis.^5^ In a retrospective evaluation of clinical trials, the frequency of moderate to severe diaper dermatitis declined by 50% with the introduction of new absorbent materials.^6^

Exclusively breastfed infants have stool that is well known to be difficult to contain.^7^ Because of the heterogeneous consistency of breastfed infant stool, the stool is often not sufficiently absorbed into the core of the diaper and may be in contact with the skin. Because the stool is not adequately contained, it can leak out of the diaper, where it can increase the risk of skin irritation and dermatitis outside of the diapered region. Compounding this issue, exclusively breastfed infants have a higher median stool frequency per day than infants who are fed formula.^8^

The frequency and unique consistency of infant stool for exclusively breastfed infants presents specific challenges that traditional diaper offerings may not adequately address, prompting the development of a new diaper offering. A diaper design that can more effectively lock away breastfed infant stool represents a valuable tool that can aid in the mitigation of diaper dermatitis. Presented in this research are two studies that investigated the ability of a diaper with a meshlike aperture topsheet to absorb the stool of breastfed infants. Excerpts of these studies were presented at National Advanced Practice Neonatal Nurses Conference, San Diego, CA, 2016.^7^

## 2 MATERIALS AND METHODS

**Figure 1:**
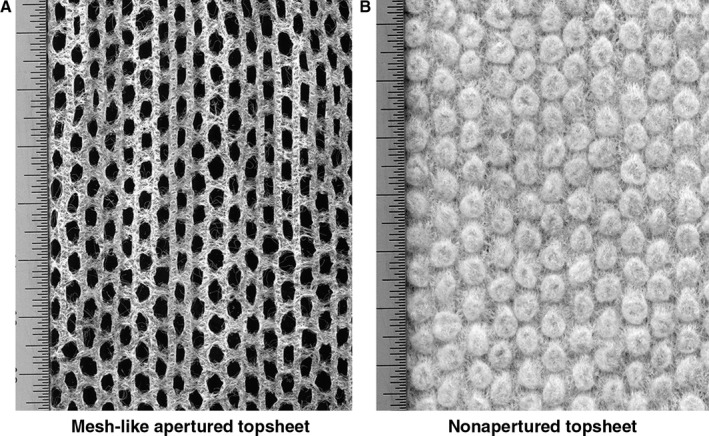
Topsheet scan images. A, Meshlike apertured topsheet. B, Nonapertured topsheet

Two types of commercially available diapers were evaluated in an at‐home diaper evaluation diary and in a benchtop test; one had a meshlike apertured topsheet to allow penetration of stool to the interior of the diaper (Figure 1A), while the other had a nonapertured topsheet designed to immobilize stool in the topsheet (Figure 1B). Detailed materials and methods for each study are available as supplemental materials to this manuscript.

### 2.1 Laboratory trans‐topsheet capacity study design

Trans‐topsheet capacity (TTSC) relates to the ability of breastfed stool to readily penetrate an absorbent structure under a small applied pressure. This benchtop study was conducted using two types of fresh stool samples (watery and mucousy) from exclusively breastfed infants using previously employed methods.^7^

### 2.2 At‐home diaper evaluation diary design

The comparative absorbency of the diapers was evaluated by looking at the ability of the diapers to keep feces away from babies' skin, as measured by an evaluation of the amount of feces present on the babies' skin for each diaper change. In addition, the absorbency was evaluated by looking at the ability of the diapers to protect against leakage of feces.

Caregivers completed an online diary with every diaper change and were instructed to record incidence of diaper leakage and to rate the test products at each diaper change regarding the amount of stool stuck to the infant's skin or retained in the diaper using the following five‐point scale: All on the skin, Mainly on the skin, Half on skin/Half in diaper, Mainly in the diaper, and All in the diaper.

### 2.3 Data analysis

The diary data were statistically analyzed with the GLIMMIX procedure of The Statistical Analysis System (SAS) software version 9.4.

## 3 RESULTS

### 3.1 Laboratory study

Watery and mucousy breastfed infant stool absorbency was tested in size 1 diapers to represent both ends of the consistency range of breastfed stool (Table 1).

**Table 1:** Meshlike apertured diapers allow significantly more breastfed stool to pass through the topsheet

Stool type	Product	Trans‐topsheet capacity (g/square inches)	*P* Value
Mucousy	Meshlike apertured	0.76	< 0.0001
	Nonapertured	0.19	
Watery	Meshlike apertured	1.84	< 0.0447
	Nonapertured	1.54	

The number of samples was at least eight per product per condition. There was a significant increase in the TTSC values of both mucousy (0.76 g/square inches) and watery (1.84 g/square inches) stool for the meshlike apertured design compared to the mucousy (0.19 g/square inches) and watery (1.54 g/square inches) stool for the nonapertured design indicating better absorption of the stool by the aperture topsheet (*P* < 0.05).

**Figure 2:**
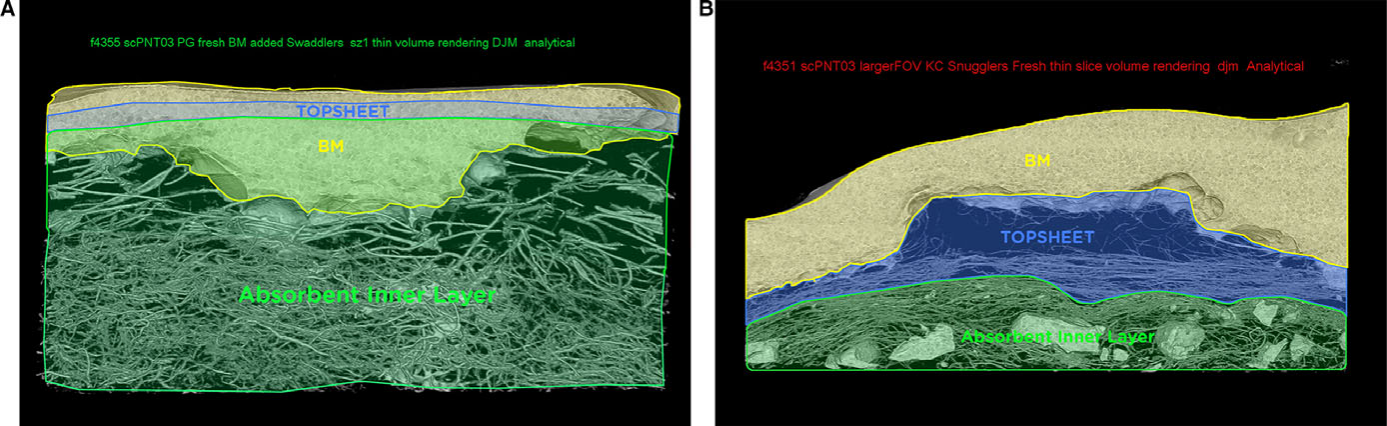
CT images of stool penetration below topsheet into absorbent core. A, Meshlike apertured diaper. B, Nonapertured diaper

As verified by computed tomography (CT) images, in the meshlike apertured design, stool penetrates past the topsheet and is held in the absorbent layer below (Figure 2A). The nonapertured design reveals that a substantial amount of stool remains on the topsheet and is not drawn away into the diaper (Figure 2B).

### 3.2 At‐home diaper evaluation diary

To further assess performance between the two types of diapers for exclusively breastfed infants, the percentage of stool all or mainly in the diaper at the time of diaper change was examined. Of the 354 survey participants, 86 of the infants were exclusively breastfed (Table 2). For infants fed only breastmilk, a statistically significant increase in diaper changes with all or most of the stool absorbed in the meshlike apertured diaper (58.6%) vs the nonapertured diaper (48.9%) was seen (*P* = 0.0154). In addition, the study showed that breastfed infants experienced a lesser percentage of stool all or mainly on the skin with the meshlike aperture diaper (3.6%) compared with the nonapertured diaper (5.8%) (*P* = 0.18).

**Table 2:** Diaper performance for exclusively breastfed infants

Type of diaper	Number of infants	Number of diapers	Stool all or mainly on skin		Stool half on skin/half in diaper		Stool all or mainly in diaper (ie; away from skin)	
			Percent of diaper changes	*P* value	Percent of diaper changes	*P* value	Percent of diaper changes	*P* value
Meshlike apertured	86	696	3.6%	0.18	37.8%	0.023	58.6%	0.015
Nonapertured	86	677	5.8%		45.3%		48.9%	

In addition to providing a benefit related to keeping stool off the skin, the meshlike aperture diaper offered the benefit of fewer instances of stool leaking outside of the diapered area (Table 3). Of the nearly 700 diapers used by 86 exclusively breastfed infants, a statistically significant decrease in leakage rate in the meshlike apertured diapers (14.5%) vs the nonapertured diapers (21.8%) was noted (*P* = 0.0021).

**Table 3:** Real‐world stool containment consumer diary testing for exclusively breastfed infants

Type of diaper	Number of infants	Number of diapers	Stool leakage	
			Percent of diaper	*P* Value
Meshlike apertured	86	696	14.5%	0.002
Nonapertured	86	678	21.8%	

## 4 DISCUSSION

Modern disposable diapers are high‐performance products with carefully designed layers and liners to provide optimal urine and feces absorption combined with a comfortable fit. Technological advancements such as polymers and new topsheet materials can provide optimal absorption of feces, thereby minimizing skin exposure.^9^

Diaper design has long been a way to manage and prevent diaper dermatitis. The introduction of absorbent gels in diapers has been associated with a significant reduction in the severity and incidence of irritant diaper dermatitis.^6^ One of the most common irritants causing dermatitis is fecal proteases and lipases, which can weaken the physical integrity of the skin.^10^ Because of the key role that diapers can play in mitigating diaper dermatitis, many physicians recommend the use of super‐absorbent diapers, which can decrease the incidence and severity of diaper dermatitis.^11^

Infant stool presents a wide variety of consistencies. Alterations in skin pH can lead to a breakdown in skin barrier function, eventually causing diaper dermatitis.^12^ When urine and stool are left in contact with the skin, the breakdown of urea in the presence of fecal urease can increase the pH of the diaper environment. The rise in pH increases the activity of fecal proteases and lipases, which can damage skin.^10^ By reducing the contact between skin and feces, the risk of skin irritation and diaper dermatitis is reduced.

Using both laboratory and in‐use methodologies, the performance of a diaper with a meshlike apertured topsheet design was compared to a diaper with a nonapertured topsheet for breastfed infants. Laboratory testing confirmed that the percentage of stool within the diaper's core was significantly increased for the meshlike apertured design vs the nonapertured design.

The diary analysis demonstrated that the meshlike aperture topsheet design, among exclusively breastfed infants, can significantly reduce the amount of stool on the skin for exclusively breastfed infants. In addition, the apertured design was significantly better for leak prevention among breastfed infants.

## 5 CONCLUSION

It is proposed that the meshlike apertured topsheet diaper design is superior to the nonapertured design for handling infant stool, in particular hard‐to‐contain breastfed infant stool, because in both laboratory and in‐home testing, it allowed stool to pass through the topsheet and be absorbed in the diaper core better than the nonapertured design. More efficient containment and absorption of stool and the resultant reduction in stool contact with the skin may help manage the diaper conditions that can lead to skin irritation and promote better infant skin health. In addition, the aperture topsheet diaper design can reduce the number of leakages, providing added convenience for caretakers.

## CONFLICT OF INTEREST

All authors are full‐time employees at Procter & Gamble, who funded this research through an educational grant.

## STATEMENT OF INSTITUTIONAL BOARD APPROVAL/INFORMED CONSENT

For infants participating in stool collection, parental consent was given to participate in the study.
