# Supplementary material for: The impact of diaper design on mitigating known causes of diaper dermatitis
Source: Pediatr Dermatol. 2018 Aug 30;35(6):792–5. doi: 10.1111/pde.13680 (PMC6282588; doi:10.1111/pde.13680)
Supplement: Supplementary file 1 [file PDE-35-792-s001.docx]

**SUPPLEMENTAL MATERIALS**

**MATERIALS AND METHODS**

The research presented herein was an observational study. The first study was in a laboratory setting to quantify the amount of stool that could be contained and the second was an at-home study that recorded observations during each diaper change.

*Test Diapers*

Two types of commercially available diapers were evaluated in an at-home diaper evaluation diary and in a benchtop test; one had a mesh-like apertured topsheet to allow penetration of stool to the interior of the diaper (Figure 1A), while the other had a non-apertured topsheet designed to immobilize stool in the topsheet (Figure 1B).

*Laboratory Trans-Top Sheet Capacity Study Design*

The benchtop study was conducted using two types of fresh stool samples (watery and mucousy) from exclusively breastfed infants using previously employed methods,^1^ stool was collected into sterile pediatric U-bags (Briggs Healthcare Supplier #07511) that were attached around the anus.

Separate pediatric collection bags (Briggs Healthcare Supplier #07511) were also attached to the genital area of the infant to collect and keep urine separate while avoiding contamination of the stool samples. A modified diaper was applied over the pediatric collection bags, which had a portion of the core removed and a clear plastic bag attached to the back to allow room for the collection bags to expand while being filled.

Measuring trans-topsheet capacity (TTSC) is a methodology used to assess the amount of stool absorbency, on a unit area basis, within the diaper by determining the amount of stool able to pass through the topsheet (in a z-direction under pressure at a defined time and area and absorbed by the layers underneath). TTSC relates to the ability of breastfed stool to readily penetrate an absorbent structure under a small applied pressure. While this laboratory method does not simulate all the factors in a usage situation the TTSC data clearly shows differences between the tested products that supplement, and support, the in-use data. The method and equipment are described in patent 5,941,864.

In the testing described below, samples consisted of exclusively breastfed infant stool. The apparatus used to obtain TTSC data was a stainless steel cylinder mounted on a plate, with a height of 2.95 inches, an inside diameter of 2 inches and an outside diameter of 2.48 inches. The bottom of the cylinder extended below the plate a distance of 0.138 inches and contained a lip with an annular thickness of 0.138 inches. In our setup, the lip prevented the stool from leaking outside the designated test area of the sample. The apparatus contained a 101 gram weight, which is cylindrically shaped and has a diameter of 2 inches, so that it was able to fit tightly within the cylinder but could freely slide throughout the hole in the cylinder. This arrangement provided a pressure of 49.6 kilograms per square meter and a test area of 3.14 square inches. The weight was designed with a handle to allow easy insertion and removal of the cylinder.

A 4 by 4 inch square size was cut from the center of the diapers evaluated so that the absorbent core was inside of the square, ensuring all layers and components of the diaper from the topsheet through the backsheet were included. The topsheet (or its equivalent) was removed, and the remaining diaper cross section was weighed to the nearest 0.01 grams. It was found that if difficulty was encountered in removing the cutout from the diaper, or in removing the topsheet, the diaper could be frozen prior to or after cutting, using a PH100-15 circuit refrigerant (Philips ECG, Inc. of Waltham, Mass). The topsheet or corresponding top layer was then returned to its original position, and the cylinder was centered over the square in preparation for stool sample application.

In order to obtain enough stool for TTSC testing, samples of similar consistency (mucousy or watery) were combined together with gentle stirring using a metal spatula. Care was taken to minimize mixing in order to prevent breakdown of the stool’s consistency. These mixtures also provided consistency across testing, as the same sample (mucousy or watery), was tested across the different diaper samples. TTSC testing was conducted at room temperature. A syringe dispensed 9 grams of stool through the hole in the cylinder onto the top of the diaper cutout. The 101 gram weight was pushed through the hole in the cylinder to ensure equal distribution of the stool onto the topsheet. After a period of 2 minutes, the apparatus and topsheet were removed and the remainder of the sample was reweighed to assess the amount of stool that passed through the topsheet into the absorbent core. The difference in weight of the layers below the topsheet represented how much stool penetrated through the topsheet into the layers below, divided by the cutout 3.14 square inches. TTSC values are reported in grams/square inches.

Computed tomography (CT) images were obtained via a small sample of the diaper core that was prepared using a punch tool. The sample was carefully transferred into a CT sample holder and placed between Styrofoam rings under slight pressure from a retaining disk and then sealed with parafilm to prevent fluid loss. Measurements were started approximately 1 hour or more after sample preparation to allow for equilibration. Each CT dataset took approximately 6 to 8 hours to collect.

*At-Home Diaper Evaluation Diary Design*

The purpose of this study was to evaluate the relative performance of two commercially available diapers, one with a mesh-like aperture topsheet and one with a non-apertured design. The comparative absorbency of the products was evaluated by looking at the ability of the diapers to keep feces away from babies’ skin, as measured by an evaluation of the amount of feces present on the babies’ skin for each diaper change. Additionally, the absorbency was evaluated by looking at the ability of the diapers to protect against leakage of feces.

The at-home diaper evaluation diary was a blinded, cross-over comparison test with a technical diary. Panelists used two products alternately in a 2 sequence rotation (AB/BA) paired comparison protocol with the requirement that each panelist complete a diary to assess the amount of feces present on the skin and leakage protection.

The study consisted of 365 infants aged 0 to 6 months in 11 geographically dispersed markets throughout the United States who were bottle-fed, breastfed or consumed a mixed diet. In each market, respondents were randomly split into 2 groups, each with one of the 2 product rotations. The study took place between July 9, 2014 and August 9, 2014. Diapers were removed from their original packaging and rewrapped in plain white packaging for blinding purposes.

Caregivers completed an online diary with every diaper change. Each panelist was instructed to use each product for 5 days/nights each as they normally would use their regular diapering products both during the day and overnight. Panelists were instructed to use the products in the order as labeled and to complete the online diary questionnaire for each diaper after each diaper change. If diapers only contained urine, and no feces, it was excluded from analysis. If the panelists ran out of diapers for one product before the 5 days/nights, panelists were instructed to then proceed to use the next product. All links were provided to the panelists via email.

Panelists were instructed to fill in the product code on the package label with every online diary entry. Panelists were also provided with diary back-up sheets if the online questionnaire was inaccessible to the panelists. Panelists were instructed to enter the information from the backup sheet once the online questionnaire became accessible again. Caregivers were instructed to record incidence of diaper leakage and to rate the test products at each diaper change regarding the amount of stool stuck to the infant’s skin or retained in the diaper using the following five-point scale: All on the skin, Mainly on the skin, Half on skin/Half in diaper, Mainly in the diaper and All in the diaper. Eleven subjects were excluded from the analysis as a stool diaper change was not reported for both the mesh-like aperture diaper and the non-apertured diaper.

*Data Analysis*

The diary data was statistically analyzed with the GLIMMIX procedure of The Statistical Analysis System (SAS) software version 9.4. In each case, a generalized estimating equation model for a binomial distribution and logit link function were fitted to the data. The performance of the two diaper designs was compared to determine whether differences were statistically significant.

**REFERENCES**

1. Kenneally, D., Gustin, J., Roe, D., Waimin Siu, S., & Kutay, B. (2016, April). Understanding the characteristics of breastfed baby stool to help better manage runny bowel movements. Poster session presented at the National Advanced Practice Neonatal Nurses Conference, San Diego, CA.
